# Assessment of Awareness and Knowledge about Osteoporosis in Relation to Health Prevention among Patients Treated in Osteoporosis Clinics

**DOI:** 10.3390/jcm12196157

**Published:** 2023-09-24

**Authors:** Agnieszka Barańska, Bartłomiej Drop, Urszula Religioni, Joanna Dolar-Szczasny, Maria Malm, Krystian Wdowiak, Magdalena Bogdan, Anna Kłak, Piotr Merks, Robert Rejdak

**Affiliations:** 1 Department of Medical Informatics and Statistics with e-Health Lab, Medical University of Lublin, 20-059 Lublin, Poland; bartlomiej.drop@umlub.pl (B.D.); maria.malm@umlub.pl (M.M.); 2 School of Public Health, Centre of Postgraduate Medical Education of Warsaw, 01-813 Warsaw, Poland; urszula.religioni@gmail.com; 3 Department of General and Pediatric Ophtalmology, Medical University of Lublin, 20-059 Lublin, Poland; joannaszczasny@op.pl (J.D.-S.); robert.rejdak@umlub.pl (R.R.); 4 Faculty of Medicine, Medical University of Lublin, 20-059 Lublin, Poland; krystianrwdowiak@interia.eu; 5 Department of Social Medicine and Public Health, Warsaw Medical University, 02-007 Warsaw, Poland; mbogdan@wum.edu.pl; 6 Department of Environmental Hazards Prevention, Allergology and Immunology, Medical University of Warsaw, 02-091 Warsaw, Poland; anna.klak@wum.edu.pl; 7 Department of Pharmacology and Clinical Pharmacology, Faculty of Medicine, Collegium Medicum, Cardinal Stefan Wyszyński University, 01-815 Warsaw, Poland; p.merks@uksw.edu.pl

**Keywords:** awareness, knowledge, osteoporosis, risk factors, osteoporosis patients

## Abstract

The increasing incidence of osteoporosis indicates that the disease is a serious public health problem, with about 200 million people being affected worldwide. The aims of this research are to assess the awareness and knowledge about osteoporosis in relation to risk factors, health condition, supplementation used, socio-demographic factors and other variables among osteoporosis patients. The study was conducted in 2016–2018 in osteoporosis clinics in Poland. The study involved 312 patients with a diagnosis of osteoporosis. In the diagnostic survey method, the authors’ own questionnaire was used. The results indicate that the more frequent the symptoms associated with the disease, the lower the general self-assessment of the health condition of the respondents (rho = −0.682, *p* < 0.001). In addition, almost half of the respondents stated that their knowledge of osteoporosis is negligible. Moreover, the use of dietary supplements significantly differentiated respondents in terms of health self-assessed (*p* < 0.001), and it is noteworthy that users of dietary supplements assessed their health significantly better. We also saw a statistically significant relationship between the self-assessment of knowledge about osteoporosis and the use of dietary supplements (*p* < 0.001). Accordingly, significantly more respondents rating their knowledge as good or very good used dietary supplements. The conducted study demonstrates the need to educate patients and implement educational programs at central and provincial levels to improve patient knowledge concerning the disease. Supporting adaptation to chronic diseases and appropriate therapeutic management may contribute to improved osteoporosis treatment and enhanced patient quality of life.

## 1. Introduction

Osteoporosis is currently a major health and socioeconomic problem [1,2,3]. Epidemiological data indicate that osteoporosis is one of the most frequent chronic diseases, with about 200 million people being affected worldwide, including every third post-menopausal woman, as well as most elderly people over 70 years of age [4].

Osteoporosis is often asymptomatic, causing painless deterioration of the skeletal system through progressive destruction of bone mass. A noticeable symptom of the disease, however, is usually an osteoporotic fracture, indicating advancement of the disease [5,6]. One of the divisions of osteoporosis distinguishes between two types: primary and secondary osteoporosis [7]. 

Primary osteoporosis includes idiopathic and involutional osteoporoses. Idiopathic osteoporosis occurs in children and adolescents, and leads to fractures [8,9]. Suggested causes involve endocrine basis, vitamin D deficiency, an infectious agent, immunological disorders and genetic factors [10,11,12]. The second type of primary osteoporosis, the most frequent, is involutional osteoporosis. This is closely related to the aging process, when the bone metabolism processes are disturbed and, as a consequence, the appearance of osteopenia is accelerated [13,14]. Involutional osteoporosis has been divided into two types. The first type is post-menopausal osteoporosis due to estrogen deficiency, and it mainly affects the spongy bone [15,16]. The second type is senile osteoporosis, which involves the loss of bone mass due to the aging of the cortical and spongy bone [17,18,19]. Primary osteoporosis most commonly develops in postmenopausal women (~25%) and less commonly in elderly men [20]. The following factors are considered to play a key role in the occurrence of osteoporosis: genetic factors, hormonal balance, diet, physical activity and care for health during the growth process [21,22,23,24].

Secondary osteoporosis is caused by factors other than the natural aging and menopausal processes [25]. It can result from taking certain groups of medications, which include glucocorticosteroids, anti-epileptic drugs, heparin, thyroid hormones, aluminium-containing gastric suppressants, sedatives, oral anticoagulants, antituberculosis drugs, chemotherapeutics, tetracyclines and diuretics [26,27]. The above-mentioned medications increase the risk of developing the condition, and iatrogenic secondary osteoporosis caused by chronic glucocorticosteroid use is considered the most common secondary osteoporosis [28,29]. The appearance of secondary osteoporosis can also be favored by certain diseases, for example hyperthyroidism, hyperparathyroidism, rheumatic diseases, diabetes, and malabsorption syndrome [30,31,32,33]. 

Age is the dominant risk factor for osteoporotic fractures [34,35]. It is estimated that approximately 90% of all bone fractures occur in patients over the age of 60 [36,37]. In the perspective of an aging population, it becomes important to take action to prevent the condition and to raise health awareness of osteoporosis risk factors.

The aim of this research is to assess the awareness and knowledge about osteoporosis in relation to risk factors, health condition and factors that have the greatest impact on the health condition of people suffering from osteoporosis. Accordingly, a detailed analysis of the above aspect includes correlations regarding the frequency of ailments related to the disease, supplementation used and socio-demographic variables.

## 2. Materials and Methods

The study was conducted in 2016–2018, in five osteoporosis clinics in the Lubelskie macroregion, in eastern Poland. The study involved 312 patients with a diagnosis of osteoporosis. Inclusion criteria established prior to the start of it included patients who were treated for osteoporosis for at least one year who were 45 years of age or older and provided informed consent to participate in the study. The study design was cross-sectional and was a part of a scientific project carried out in 2016–2019 in the Lubelskie macroregion for patients treated for osteoporosis. The research method was a diagnostic survey in which a self-designed questionnaire was used. It was created according to the survey method commonly employed for analyses of the relationships between variables. This questionnaire included questions about sociodemographic data, health self-assessment, supplementation, knowledge about osteoporosis and osteoporosis prevention, comorbidities, ailments, treatment time and methods used for osteoporosis diagnosis. After a pilot study, the questionnaire was evaluated and modified.

Selection of respondents was random and purposive. The indispensable element of randomness was introduced by inviting every second applicant for the study. Based on the estimations (the study population for Poland is approximately 4 million and the sample size is 312), appropriate calculations were performed associated with the selection of the sample. Assuming a confidence interval of 95%, the maximum error was 6%. This means that with an only 5% risk of error, it may be estimated that a given result in the examined population may reflect a figure 6% higher or lower than for the total population.

### 2.1. Ethical Issues 

This study was performed in line with the principles of the Declaration of Helsinki. Participants were informed of the purpose and anonymity of the study being conducted and provided written informed consent to participate. Approval was granted by the Ethics Committee Medical University of Lublin (decision nr:KE-0254/175/2016).

### 2.2. Statistical Analysis

Analysis of results was performed using Statistica v.13 software (StatSoft, Cracow, Poland). Continuous variables were reported as means (M) ± standard deviation (SD), and minimum (MIN)–maximum (MAX) range. Mann–Whitney U test, Spearman correlation coefficient and Chi-2 test were employed to determine the relationship between variables. Before the tests were applied, assumptions on the normality of distribution were verified by means of the Shapiro–Wilk test.

## 3. Results

The study included 312 respondents treated for osteoporosis. The data imply the respondents were in the age range of 45–88 years old. The average age of the study group was 62.76 years ± 9.13. The vast majority were women (91.7%, n = 286). Men accounted for the remaining 8.3% of the study group (n = 26). Most of the respondents lived in the city (61.5%, n = 192), while 38.5% lived in the countryside (n = 120). The study group was dominated by people with vocational (27.6%, n = 86), secondary (25%, n = 78) and higher (22.1%, n = 69) education.

On analysis of current employment status in the study group, it was found out that slightly over half of all respondents (56.1%, n = 175) were retired people and pensioners. Almost every fourth respondent (22.4%, n = 70) was a white-collar worker, while the least numerous group consisted of manual workers (16.3%, n = 51) and the unemployed (5.1%, n = 16). The criterion for inclusion in the study was treatment for osteoporosis for more than 1 year. More than half of the respondents were treated for osteoporosis from 1 to 5 years (62.9%). In turn, 27.6% of all patients were under treatment for 6 to 10 years. The least numerous group (9.6%) were people suffering from osteoporosis for more than 10 years. 

Most of the surveyed were diagnosed with at least one additional disease (apart from osteoporosis). The greatest number of respondents indicated that they had type I diabetes (26.3%) and rheumatoid arthritis (24%), while every tenth examined person suffered from chronic liver disease. Hypogonadism or premature menopause before the age of 45 were indicated by 6.7% of the respondents, and similarly untreated long-term hyperthyroidism—by 6.1% of the respondents. Other conditions (congenital bone fracture, chronic malnutrition or malabsorption syndrome, other chronic diseases) were significantly less frequent. 

Half of the respondents indicated that the onset of osteoporosis was influenced by their age over 50 years (50.5%). Nearly half considered calcium deficiency in the body as the cause of the disease (46.6%). There were also many people indicating the consumption of large amounts of tea and coffee (42.4%), family predispositions (40.8%), excessive physical effort (38.8%), small body build (36.9%), smoking (36.9%) and vitamin D3 deficiency (32.4%).

Slightly more than half of the respondents assessed their health condition as good (56.4%). However, a significant percentage of patients also rated their health as bad (38.5%). Only a few respondents indicated that their health was very good (3.8%) or very bad (1.3%).

Self-assessment of health is negatively and very strongly correlated with the frequency of osteoporosis-related symptoms (rho = −0.682, *p* < 0.001). The more frequent the symptoms associated with the disease, the lower the general self-assessment of health condition of the respondents. The trend is clear and very strong. It is also worth noting that people with daily symptoms assessed their health significantly worse. This included the largest percentage of respondents, who indicated that the ailments associated with osteoporosis occur every day (Table 1).

Use of (any) dietary supplements significantly differentiated respondents in terms of self-assessed health (Z = −1.638, *p* < 0.001). It is noteworthy that users of dietary supplements assessed their health significantly better, as good (57.3%) or very good (4.4%).

Almost half of the respondents stated that their knowledge of osteoporosis is negligible (47.4%). Every third respondent assessed it as good (33.3%), while 13.8% determined their knowledge as very good, and the remaining 5.5% of respondents declared a complete lack of knowledge about osteoporosis.

When asked about sources of knowledge about osteoporosis, respondents most often pointed to healthcare professionals (28.5%). For many respondents, mass media (24.7%) and books/magazines (21.2%) were sources of information. One in ten respondents gained knowledge of the disease from friends (10.9%) or other sick people (6.4%) and a small proportion of respondents declared that they were not interested in the subject (8.3%).

There was a statistically significant relationship between the self-assessment of knowledge about osteoporosis and the sources from which the respondents obtained knowledge on this subject (*p* < 0.001). The analysis shows that the respondents assessing their knowledge as good or very good most often derived it from healthcare professionals (49.7%), and less often from books/magazines or mass media (27.9%).

There was a statistically significant relationship between the self-assessment of knowledge about osteoporosis and the use of dietary supplements (*p* < 0.001). Accordingly, significantly more respondents rating their knowledge as good or very good (98%) used dietary supplements.

There were statistically significant relationships between the self-assessment of knowledge about osteoporosis and the use of certain dietary supplements (correlations at *p* < 0.001). All the analyzed categories of supplements were taken by statistically significantly more people assessing their knowledge about osteoporosis as very good or good than by those assessing their knowledge as negligible or declaring its complete lack (Table 2).

There was a statistically significant relationship between the assessment of the state of knowledge about osteoporosis and the assessment of the financial situation of the respondents (*p* < 0.001). The vast majority of people who assessed their financial situation as very good declared very good or good knowledge about osteoporosis (91.9%). 

The analysis showed that there was a statistically significant relationship between the self-assessment of knowledge about osteoporosis and the respondents’ level of education (*p* < 0.001). The vast majority of respondents with higher education declared very good or good knowledge about osteoporosis (95.7%). In contrast, only half of respondents with secondary education, one in three with post-secondary education (32.7%), and only one in five with primary (23.3%) or lower secondary (20%) education had knowledge at this level.

Statistical analysis showed that respondent sex (*p* < 0.072) and age (*p* < 0.522) did not affect self-assessment of knowledge about osteoporosis. There was also no relationship with place of residence (Z = −0.835, *p* < 0.455).

## 4. Discussion

The study showed that most patients diagnosed with osteoporosis also suffer from other chronic diseases, with diabetes and rheumatoid arthritis being the most common comorbidities in the studied population. This finding is consistent with the results of numerous scientific papers that have found an association between osteoporosis and hypertension [38], diabetes [38,39], metabolic syndrome [40] and rheumatic diseases [41].

In a study by Janiszewska et al., the respondents most frequently indicated older age (89.4%), menopause (85.4%) and a diet low in calcium (77.6%) as risk factors for osteoporosis [42]. In the Lebanese population, older age (81.8%), a diet low in calcium (81.8%) and vitamin D3 deficiency (79.3%) were most commonly indicated [38]. In a study by Hammoudeh et al., low calcium intake (96.7%), older age (89.5%) and a family history of osteoporosis (86.0%) were the most commonly selected [43]. Egyptian respondents most frequently indicated female gender (48.4%), older age (29.8%) and low calcium content in the diet (5.7%) [44]. In our own study, the following were most often indicated: older age (50.5%), calcium deficiency in the diet (46.6%) and consumption of large amounts of tea and coffee (42.4%), while menopause was relatively rarely associated with this disease (22.3%). Based on the above results, it can be concluded that osteoporosis is associated in society with being elderly and following a diet low in calcium. A relatively small percentage of respondents also associated cigarette smoking and caffeine consumption with osteoporosis [38,42,44].

Dietary supplements containing calcium and vitamin D have been the subject of numerous studies that have shown their positive effect on bone structure [45,46,47]. In contrast, magnesium supplementation remains a controversial issue due to reports of negative effects of higher doses of this macronutrient on bones [45,48,49]. On the basis of our own research, we found that patients with osteoporosis who use supplements better assessed their health and knowledge about the disease. These individuals most often used calcium and phosphorus preparations (71.2%), vitamin D (70.5%) and magnesium (37.8%). In other studies, calcium was most often supplemented, and the percentage of people supplementing it is also increasing over time [38,46,50].

Based on the conducted study, it was found that the main source of information on osteoporosis for the respondents are healthcare professionals and the mass media. The first group felt better informed. On the basis of studies by Ahmadieh et al. [38], Hammoudeh et al. [43] and Ribeiro et al. [50], the same conclusions can be drawn. In view of the above, attention should be paid to the need to increase the education of patients by healthcare workers. Osteoporosis prevention is becoming more important in the face of the progressive aging of society. Despite the significant impact of osteoporosis on the health and quality of life of older people, many medical entities lack knowledge about the issue of osteoporosis, which leads to insufficient patient care [51]. Understanding the factors that influence preventive measures is of key importance for the prevention of osteoporosis at the population level. 

Limitations of the study should be indicated. The first may be the lack of access to patients’ medical records; it would allow for a detailed analysis of other medical aspects and their possible impact on the health status and self-assessment of patient knowledge. The second may be the lack of classification of patients according to the severity of osteoporosis, the assessment of fractures and the therapies used. These limitations warrant further research in chronic diseases such as osteoporosis.

## 5. Conclusions

The analysis of the awareness and knowledge of patients treated for osteoporosis indicates that a very important aspect in the prevention of osteoporosis is the health education of patients. Self-assessment of knowledge about the disease significantly differentiated the intake of dietary supplements and was associated with sociodemographic characteristics such as respondent education and financial situation. A factor influencing respondent ability to function was their individual perception of their own health related to the frequency of pain complaints. The conducted study demonstrates the need to educate patients and implement educational programs at central and provincial levels to improve patient knowledge concerning the disease. Supporting adaptation to chronic diseases such as osteoporosis and appropriate therapeutic management may contribute to improved osteoporosis treatment, reduced pain and enhanced patient quality of life.

## Figures and Tables

**Table 1:** Frequency of osteoporosis-related symptoms and self-assessment of health (Spearman correlation coefficient).

How Do You Assess Your General Health Condition?	How Often Do You Notice the Occurrence of Ailments Related to Osteoporosis?										In Total	
	Do Not Occur		Quite Rarely		A Few Times a Month		A Few Times a Week		Every Day			
	N	%	N	%	N	%	N	%	N	%	N	%
Very bad	0	0.0%	0	0.0%	0	0.0%	0	0.0%	4	3.4%	4	1.3%
Bad	0	0.0%	1	7.7%	4	5.6%	22	20.2%	93	79.5%	120	38.5%
Good	1	100.0%	8	61.5%	60	83.3%	87	79.8%	20	17.1%	176	56.4%
Very good	0	0.0%	4	30.8%	8	11.1%	0	0.0%	0	0.0%	12	3.8%
In total	1	100.0%	13	100.0%	72	100.0%	109	100.0%	117	100.0%	312	100.0%

N, number. Spearman rho: rho = −0.682, *p* < 0.001.

**Table 2:** Frequency of osteoporosis-related symptoms and self-assessment of health (Chi-2 Test).

Used Dietary Supplements	How Do You Assess Your Knowledge about Osteoporosis?				In Total		Chi-2 Test	
	(Very) Good		Negligible/Lack					
	N	%	N	%	N	%	Chi-2	*p*<
Vitamin D	127	86.4%	93	56.4%	220	70.5%	33.720	0.001
Magnesium	78	53.1%	40	24.2%	118	37.8%	27.455	0.001
Calcium and phosphorus preparations	125	85.0%	97	58.8%	222	71.2%	26.091	0.001
Vitamin A	30	20.4%	11	6.7%	41	13.1%	12.861	0.001
Vitamin K	37	25.2%	13	7.9%	50	16.0%	17.272	0.001
Vitamin C	68	46.3%	40	24.2%	108	34.6%	16.649	0.001
Calcitonin	34	23.1%	13	7.9%	47	15.1%	14.131	0.001
Estrogen	16	10.9%	4	2.4%	20	6.4%	9.275	0.001

N, number.

## Data Availability

Not applicable.
